# The Implementation of Governance Attributes in Health in Uasin Gishu County, Kenya

**DOI:** 10.24248/EAHRJ-D-18-00021

**Published:** 2018-11-23

**Authors:** Jackline Sitienei, Mabel Nangami, Lenore Manderson

**Affiliations:** a Center of Health Policy, School of Public Health, University of the Witwatersrand, Johannesburg, South Africa; b Health Policy and Management Department, School of Public Health, College of Health Sciences, Moi University, Eldoret, Kenya; c Institute at Brown for Environment and Society, Brown University, Providence, RI, USA

## Abstract

**Background:**

Globally, good governance is increasingly recognised as an important factor in health systems. Governance is a key determinant of performance, particularly towards achieving targets that ultimately affect economic and social development. However, conceptually and practically, governance is poorly understood by decision makers at various levels. Governance is also difficult to measure, but it is critical in assessing responsive, inclusive, effective, and efficient services. We examined the extent to which governance attributes have been implemented within the Department of Health in Uasin Gishu County, Kenya.

**Methods:**

A cross-sectional research design was adopted, with 108 decision makers forming the target population. The study period was between April and July 2016. Select documents relating to governance were reviewed; subsequently, data were collected using a self-administered, semi-structured questionnaire, with 5-point Likert-type questions and open-ended questions. We calculated proportions related to agreement levels to establish the decision makers' perceptions on the implementation of governance attributes. Cronbach's *α* for the items was between 0.72 and 0.84. Qualitative data were coded and categorised using a framework approach.

**Results:**

Of the 93 decision makers who responded, most (n=64, 68.8%) had been in their current position for less than 5 years. Regarding governance attributes, over half of the participants agreed on the implementation of good governance in terms of strategic vision as well as regulation and oversight. Around half of the participants were undecided on the implementation of good governance in terms of intelligence and information, transparency, participation, and consensus orientation. Almost two-thirds believed that accountability and equity were poorly implemented. A minority rated the overall governance score as good, while two-thirds considered governance to be poor. Corruption, nepotism, lack of transparency, political interference, and inadequate use of information were all reported to affect the implementation of good governance.

**Conclusion:**

Decision makers reported poor implementation of governance attributes at public health facilities, especially in terms of accountability, equity, community participation, consensus orientation, strategic vision, and regulation and oversight. It is feasible and critical to evaluate implementation of governance attributes to help improve governance; the successful implementation of each attribute depends on the successful implementation of all others.

## INTRODUCTION

Globally, governments are increasingly concerned with how to improve the performance of health services. Governance is increasingly considered to be a key determinant of performance,^1,2^ particularly towards achieving targets that ultimately affect economic and social development.^3–5^ Governance can be understood from a political, developmental, and health systems perspective,^3^ and it has been defined as the process of creating an organisational vision and mission; defining the goals and objectives that should be met to achieve the vision and mission; and defining the structures in that need to be place to achieve, monitor, and evaluate the performance of the desired outcomes.^4^ In the Action Plan for Universal Health Coverage, the World Health Organization defined governance as including:

**The traditions and institutions by which authority in a country is exercised for the common good, including the processes by which those in authority are selected, monitored and replaced; the capacity of the government to effectively manage its resources and implement sound policies; and the respect of citizens and the state for the institutions that govern economic and social interactions among them.^6^**

Although this definition arguably privileges organised governance structures through the agency of the state, the definition includes the involvement of civil society.^7^ Good governance is among the building blocks of any health system, yet the implementation and evaluation of governance – as well as research on governance – are often neglected at the national and international levels due to lack of clarity on operationalising governance, its complexity, and the sometimes sensitive issues that arise in determining it.^4,8^

The concern with governance in government-operated services arose from an interest in the private sector in the 1970s, when managers began to focus on the impact of governance on performance as a result of the interplay between shareholders, consumers, company executives, and boards to maximise returns.^2^ Subsequently, concern with governance was extended to consider the broader contexts – judicial, regulatory, social, and cultural – in which corporations operated. Governance in the public sector was adapted from the private sector, with the terminology of shareholders and managers replaced by that of citizens and public officers. Because the public sector is large, with a larger number of interest groups than is typical for a private corporation, the ability of the public sector to attain various goals has been seen to be diluted by contestations among various interest groups and by the vulnerability of the sector to control by those with strong interests and power.

Over the past 3 decades, due to the poor economic and social performance of many countries receiving international aid, the focus of governance in such countries has been on international development and growth to benefit citizens.^2,9–11^ At the same time, in order to fully realise the goals of development, concerns related to governance have extended to the heath sector. Investing in the governance of the health-care system is considered to be critical for the real-isation of health-care investments.^1–3,12–14^ Reporting on this necessitates being able to measure, monitor, and evaluate the implementation of governance at different levels of the system.

Although the current literature on governance has emphasised the importance of the evaluation of the implementation of governance, there is still little empirical research on this due to the complexity of governance and its role within health-care systems. A wide range of international bodies, including financial agencies and multilateral and bilateral programmes,^1^ have historically championed for sensitisation about the value of implementing good governance. These actors have proposed ways of measuring governance using different frameworks in an attempt to develop an acceptable way of measuring and monitoring governance.^1,2,4,8,15,16^ Siddiqi et al, for example, proposed 10 attributes and principles of governance: strategic vision, participation and consensusorientation, rule oflaw, transparency, responsiveness, equity and inclusiveness, effectiveness and efficiency, accountability, intelligence and information, and ethics.^4^ Ruhanen, in contrast, after analysing over 40 articles, identified the most commonly used governance processindicators tobeaccountability, transparency, involvement, structure, effectiveness, and power.^17^ These indicators serve as a guide when evaluating governance, although each attribute needs to be operationalised, and this, in turn, may vary according to local context.

In general, the attributes of governance are operationalised as follows.^1,2,4,18^ *Accountability* is arguably the strongest governance attribute, and it cuts across many other attributes. It involves answerability and the imposition of sanctions. Accountability is defined as “obligations of individuals or agencies to provide information about, and/or justification for, their actions to other actors, along with the imposition of sanctions for failure to comply and/or to engage in appropriate action.”^18^ Three types of accountability can be distinguished: financial, performance, and political accountability.^18^ There is also the distinction between internal and external accountability.^19^ Internal accountability deals with institutional bureaucratic control mechanisms, while external accountability involves mechanisms wherein the community or public hold those in public institutions answerable.^19^ *Strategic vision* provides a long-term perspective on health and development. *Participation and consensus orientation* provide a voice to the citizenry directly or through representation. *Regulation and oversight* provide the institutional and legal frameworks. *Transparency* involves the accessibility of institutional processes and information as required. *Intelligence and information* are essential for understanding how an institution or system is operating, and the decision-making processes that are involved in everyday and strategic operations. *Equity* refers to the idea that all citizens should have an equal opportunity for participation, although this may be determined through a variety of mechanisms.

Within the public sector, the drivers of governance are classified in 2 primary ways, although these are not mutually exclusive. Governance determinants or attributes – also referred to as rules-based attributes – are key processes or rules that need to exist for good governance and may include laws, regulations, procedures or similar forms of authority. Governance performance, also referred to as outcome-based indicators for measuring governance, is the expected effect or outcome of good governance,^2^ and such indicators measure the degree to which rules are being implemented.^2,20^

Siddiqi et al highlighted the problematic pathways to good governance,^4^ and yet in Kenya no identified empirical studies have been conducted on governance attributes in the health-care system at the county level, where interactions between the state and citizens are most frequent. In a systematic review to identify which governance frameworks are available and have been used to assess health system governance, Pyone et al allude to the paucity of evidence related to the assessment of governance and argue for the adoption of existing frameworks to assess governance or components of it.^21^ Of the 16 frameworks they identified from political science as well as developmental and public management, only 5 had been applied; in health-related programmes, Brinkerhoff and Siddiqi applied proposed frameworks to empirical examples.^4,18^ However, while a variety of frameworks exist that might be used to assess health systems governance, few have been applied. Given the lack of application of such frameworks and lists of good governance attributes, there is a need for research to test their practicality.

The theoretical and descriptive literature also reveals that health system governance is complex and multidimensional. No single agreed upon framework can serve all purposes; accordingly, this study sought to identify and apply frameworks that might be applicable in the context of the Kenyan health system. To define good governance, therefore, in this study we drew both from frameworks found in the literature and from what was practical in the context of the study. We defined good governance according to attributes detailed by Siddiqi – characterised by the extent to which 7 groups of attributes were implemented; these were: *strategic vision; regulation and oversight; intelligence and information; participation and consensus orientation; and equity, accountability, and transparency*.^2,22^

Kenya, like many other developing countries, strives to implement governance practices to achieve its developmental goals. Vision 2030, Kenya's long-term development blueprint, is anchored on 3 pillars: social, economic, and political.^23^ The goal of the health sector is to provide equitable, affordable, and quality services. Good health is expected to play an important role in boosting economic growth, reducing poverty, and realising social goals, such as equity and efficiency.^24^ Governance is an important factor guiding the realisation of universal health care, which is 1 of the 4 major agendas of the current Kenyan government.

In this study, information was drawn from government-published reports and guidelines and a semi-structured questionnaire. The main purpose of this study was to assess the extent to which the governance attributes, listed above, were implemented in the Department of Health in Uasin Gishu County, Kenya.

## METHODS

### Study Setting

**FIGURE 1.**
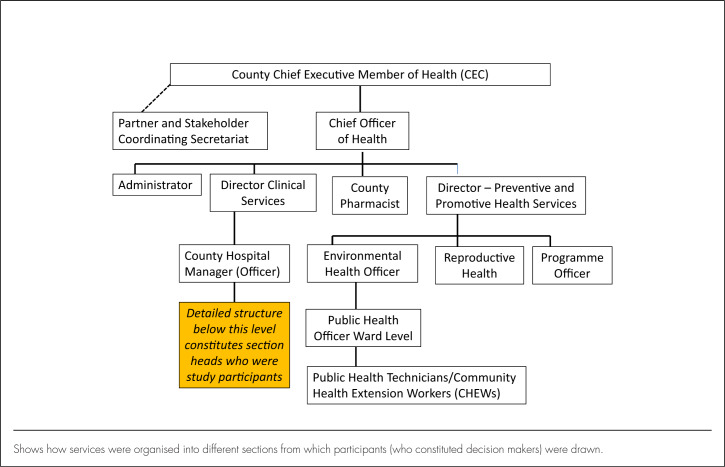
Organisational Structure of Health Services Leadership in Uasin Gishu County^25^

This study was undertaken in Uasin Gishu County, 1 of 47 counties in Kenya. The county – which has its headquarters in Eldoret, Western Kenya – has a total population of over 1 million (513,649 males and 509,292 females) according to 2016 population estimates.^25^ It is divided administratively into 6 subcounties and 30 wards.^26^ Services are organised into a 4-tier system: community, primary health care, county hospitals, and national referral hospitals.^24,27^ There are only 2 national referral hospitals – 1 in the capital city, Nairobi, and the other in Eldoret. According to the 2013 Kenya Service Availability and Readiness Assessment Mapping (SARAM) Report, there were 146 facilities providing health care, of which 90 were public primary health-care facilities.^26^ The county health service is headed by the county chief executive officer, with a deputy chief officer and 2 directors of health. Different department heads constitute the county and subcounty management teams, which participate in decision making on a range of health issues (**Figure 1**).^24^

Shows how services were organised into different sections from which participants (who constituted decision makers) were drawn.

### Study Design

We adopted a cross-sectional research design to examine the extent to which governance attributes were being implemented by decision makers in health. The cross-sectional survey design was suitable for our focus on decision makers, who belong to the constitutional level in the multilevel framework of governance (the other levels are collaborative and individual, according to Abimbola^28^), as this research design is appropriate when the data collection strategy is broader in scope and involves systematic data collection.^29^ This work was part of the first author's PhD research, looking at community participation in the governance of primary health-care facilities in the same study setting. Other aspects of governance were examined at individual and collaborative levels.

### Data Collection

Data were collected between April and July 2016. A document analysis guide (ie, a checklist used to identify governance-related material in the reviewed documents) and semi-structured questionnaires were used for data collection.

#### Desktop Review

The Uasin Gishu County Development Plan, the Uasin Gishu Strategic Plan 2013–2018, the National Health Sector Strategic Plan 2014–2018, the Kenya Health Improvement Policy, the Strategy and Plan of Action 2015–2030, and the Kenya Constitution of 2010 were reviewed for the extraction of information related to governance.

#### Survey

A semi-structured questionnaire was given to 108 constitutional-level decision makers from the county executive committee, the county health management team, and the sub-county health management team, including partners and managers of primary health-care facilities selected for the study. These were members of the Department of Health who all had the mandate to put into place the structures and processes for the implementation of good governance practices.^30,31^ The questionnaire was self-administered by all office holders willing to participate. For those whose schedules were tight, the questionnaire was delivered and collected – once completed – at a predetermined time. The first part of the questionnaire captured biographical information, and the second part was designed to elicit information on the main study objective. The second section employed a 5-point Likert-type scale and consisted of 42 statement items. These included statements like, “The objectives of the County Strategic Plan are adequate for achieving set targets”; “There are mechanisms in place to address differences in access to care by vulnerable or marginalised groups, such as women, youth, elderly, disabled”; and “The analysed data are used in planning and decision making.” On the Likert scale, 1 denoted “strongly disagree”, 2 denoted “disagree”, 3 denoted “neither agree nor disagree”, 4 denoted “agree”, and 5 denoted “strongly agree”. Open-ended, self-administered questions were used to complement the Likert-type responses, to clarify responses and provide an opportunity for participants to elaborate on particular issues. Examples of such questions included, “How is information and evidence used in decision making both at the planning and implementation levels”, “What are the issues affecting implementation of good governance practices”, and “Which are the mechanisms in place that ensure that there is equity in resource allocation and access to the health-care facility by all, including the marginalised?”

### Data Analysis

Quantitative data were cleaned and entered using Microsoft Access and exported to Stata, version 13 (Stata Corp., College Station, TX, USA) for analysis. Univariate analysis was undertaken for the demographic data, with the results presented in tables. Factor analysis was carried out for the Likert responses to explore the data before further analysis and to obtain broad explanations of the data.^32^ Varimax and Kaiser Normalisation^33,34^ were used in the factor analysis. After factor analysis, statements that had a coefficient of 0.6 and above were retained.^32^ Data were categorised into proportions to establish the extent of the implementation of governance attributes as good, undecided, or poor, and to determine an overall governance score.^32,35^

To gain a sense of the direction and extent of implementation of governance attributes, we categorised responses into 3 groups of agree, disagree, and neutral or undecided, allocating them different scores depending on the number of variables in the group. In the analysis of Likert scale responses, it is generally accepted that responses can be categorised into 2 categories, with strongly disagree, disagree and neutral treated as negative, and agree and strongly agree as positive.^36^ However, following Sullivan and Armstrong,^34,35^ we chose to summarise responses with 3 categories,^37^ with strongly agree and agree as good; undecided indicating neither good nor bad; and strongly disagree and disagree as poor, consistent with recommendations guiding researchers to expand Likert Scale response options to increase accuracy but maintain the option to condense the response range during data analysis.^32,37,38^ This allowed us to recognise efforts that reflected progress in the implementation of good governance. Governance scores were computed to obtain aggregate scores by calculating the minimum and the maximum scores.^38^

Qualitative data were cleaned, coded, and categorised into emerging themes using a framework approach. At the first level of analysis, descriptive codes were applied to gain familiarity with the emphasis that participants placed on questions of governance. Working through these broad codes, we then identified emerging themes by examining relationships and establishing linkages within and between responses, and through a process of iteration, developed primary themes. These themes were refined and finalised, with the results used to explain the findings from the Likert scale responses.

### Ethical Considerations

Ethical approval for this study was granted by the Moi Teaching and Referral Hospital, Moi University Ethics Review Committee (approval number 0001593), and University of the Witwatersrand Human Research Ethics Committee (Medical) (clearance certificate number M170497). Written permission to conduct the study was also provided by the Uasin Gishu County Department of Health. Participant consent was sought prior to the study, with all participants given an opportunity to withdraw from the study, without jeopardising their careers, if they so wished. Participants were assured of confidentiality, and the questionnaires wereallocated numeric identifying codeswithoutindicatingthenamesoftheparticipants.

## RESULTS

### Demographic Characteristics

Out of 108 questionnaires administered, 93 were completed and returned, yielding a response rate of 86%. There were approximately equal numbers of women and men, with the majority of participants being over 30 years old (**Table 1**). In terms of educational level, 67 participants had an undergraduate degree or a higher qualification, and 26 were educated no higher than the postsecondary diploma level. Most participants had been employed at their current place of work for less than 5 years. Finally, 69 participants were members of the subcounty health management team, 12 were members of the county health management team, 5 were members of the private sector, and others held positions, such as elected representative of a civil society organisation (**Table 1**).

**TABLE 1.** Demographic Characteristics of Study Participants

Variable	Category	n (%)
Sex	Male	51 (55)
	Female	42 (45)
Age group (years)	26–30	5 (6)
	31–35	6 (7)
	36–40	21 (23)
	≥41	58 (64)
Level of education	Undergraduate degree or above	67 (74)
	Diploma course and below	24 (26)
Time in current position (years)	0–5	62 (68)
	6–11	23 (26)
	≥11	5 (6)
Position	County Health Management Team	12 (13)
	Subcounty Health Management Team	69 (74)
	Private sector	5 (5)
	Other (elected representative, civil society)	7 (8)

### Desktop Review

The review of official government documentation revealed that regarding accountability, research evidence informed service delivery. A preview of the policy direction framework revealed that among other things, social accountability, participation, equity, and people-centred and efficiency principles guided service delivery in the health system. Kenya's quality model for health emphasises regulations and stake-holder involvement to enhance quality in service delivery. The Constitution of Kenya 2010, Part 2 on Rights and Fundamental Freedoms, Article 27, emphasises the need for equality and freedom from discrimination. In Chapter^6^ on leadership and integrity, public office bearers are charged with the responsibility of demonstrating respect for the people, bringing honour to the nation, dignity to the office, accountability to the public for decisions and actions, and discipline and commitment in service to the people. Chapter^12^ of the Constitution emphasises openness and accountability, including public participation in financial matters; it also emphasises that leaders should promote equitable development by making provisions for marginalised groups and areas.

Data reviewed from the Uasin Gishu County Integrated Development Plan 2013–2018 highlighted indicators for improving services and identified constant drug stock-outs as the major challenge in delivering health services, while low community involvement and limited participation in health facility governance was identified as affecting service provision. The County Health Strategic and Investment Plan detailed the roadmap on accelerating the attainment of both short-term and long-term health targets. A health system framework approach was used to define the expected outputs on leadership and governance. It encouraged public participation in preparing the county health budget and placed emphasis on stakeholder meetings, regular facility meetings, and regular meetings of the county and subcounty health management teams to monitor performance.

### Implementation of Governance

The responses to the Likert scale questions revealed that there was good implementation of 2 groups of attributes: *strategic vision* and *regulation and oversight*. Over half (n=51, 55%) of the participants reported good implementation of strategic vision, while 29 (27%) participants were neutral. Fifty (54%) participants reported good practice of regulation and oversight.

On the other hand, participants neither agreed nor disagreed on 3 groups of attributes. Almost half (n=44, 47%) of the participants were neutral (neither agreed nor disagreed) about participation and consensus orientation practices in health governance, while 39 (42%) participants disagreed.

**FIGURE 2.**
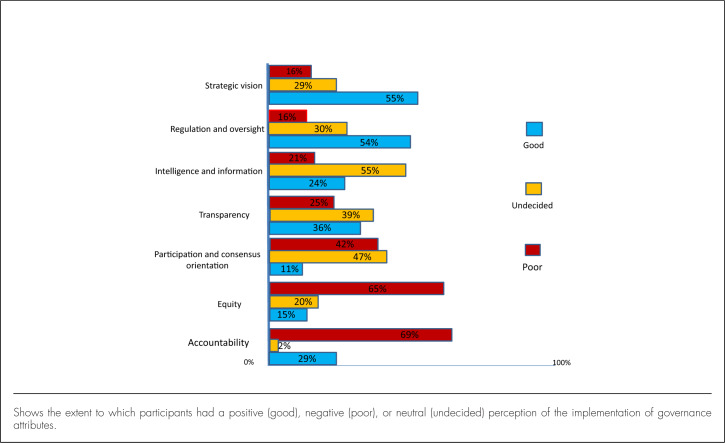
Governance Scores According to Attribute

Slightly above one-third (n=36, 39%) of the participants were neutral about practices of transparency while another third (n=33, 36%) agreed that there were good practices of transparency in county health services. However, a quarter (n=24, 25%) of the participants perceived transparency practice as poor. Over half (n=51, 55%) of the participants were neutral regarding intelligence and information. Additionally, many participants thought that implementation of governance attributes were poor in terms of accountability (n=64, 69%) and equity (n=60, 65%) (**Figure 2**).

Shows the extent to which participants had a positive (good), negative (poor), or neutral (undecided) perception of the implementation of governance attributes.

#### Governance Aggregate Score

Thirty-nine (42%) participants indicated a perception that the implementation of governance attributes was poor, while 45 (48%) were neutral, and 9 (10%) participants thought implementation of governance attributes was good.

In terms of reliability (**Table 2**), all groups of items in the tool had Cronbach's *α* values between 0.72 and 0.87, except for *accountability*, which had a Cronbach's *α* of 0.60, and this was attributed to the low number of variables in the questionnaire.^39^

**TABLE 2.** Cronbach's Alpha (*α*) Reliability Scores for Each Governance Attribute

Attribute	Items	Maximum	Median	Cronbach's *α*
Strategic vision	5	25	20	0.72
Participation and consensus orientation	8	40	25	0.82
Transparency	6	30	18	0.76
Regulation and oversight	6	30	20	0.84
Intelligence and information	8	40	26	0.75
Accountability	3	15	10	0.60
Equity	5	25	15	0.87

Findings from factor analysis yielded 7 latent factors. *Regulation and oversight* had 6 items, with the highest loading of 0.77 for the statement, “The facility managers ensure that health workers follow protocols, standards, and codes of conduct.” Two items which had been earlier placed under *intelligence and information* were also loaded in this latent factor: “The health facility collects local data,” and, “The health facility has guidelines and operating procedures for essential services from the Ministry of Health.”

Four items loaded on *intelligence and information*, with the highest, 0.75, for the statement, “Health facility managers rely on research data from the health facility to plan services.” One of the items had been earlier placed under *accountability*: “Systems exist for reporting, investigating, and adjudicating misallocation or misuse of resources (formal or informal systems)”.

Four items loaded on *strategic vision*, with the highest score, 0.77, for the statement, “Implementation of mechanisms is in line with stated objectives of health policy.” Two loaded on *participation and consensus orientation*. The highest score was in equity, with 0.90, for the statement, “There are mechanisms in place to address differences in access to care by vulnerable or marginalised groups, such as women, youth, elderly, disabled.” Other statements that scored high were, “There are structures in place to empower marginalised voices, including women, by giving them a voice in formal decision-making structures and processes” (0.84) and, “The analysed data is used in planning and decision making” (0.81).

### Qualitative Findings

The majority of participants cited nepotism, tribalism, and patronage as hindering the implementation of good governance attributes. Corruption, lack of transparency, political interference, and lack of equity in the distribution of resources were also mentioned as inhibiting good governance. Poor morale was attributed to low salaries and the delayed payment of salaries, as various participants explained:

***There is a problem in governance of our health facilities in this county.***

(Male, 36–40 years old)

***There is bias and incompetence arising from people who are not qualified being employed in positions they cannot manage.***

(Female, ≥41 years old)

***The devolution of health services at the county level has reduced morale for staff performance due to challenges of delayed salary payment and injustices in promotions.***

(Female, 26-30 years old)

**FIGURE 3.**
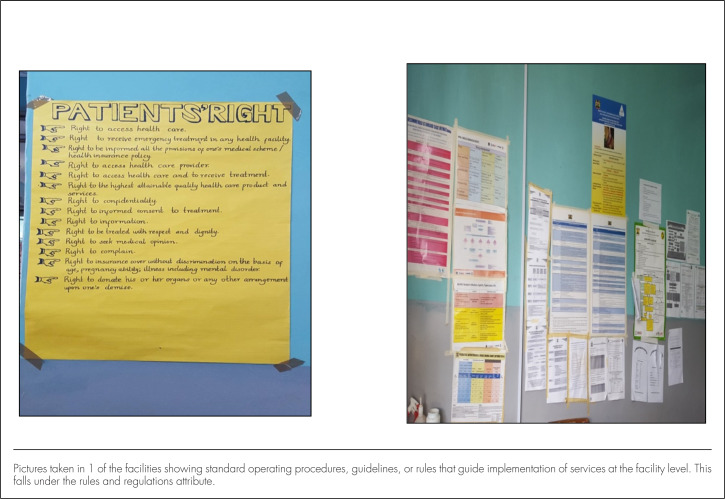
Rules and Guidelines

Pictures taken in 1 of the facilities showing standard operating procedures, guidelines, or rules that guide implementation of services at the facility level. This falls under the rules and regulations attribute.

Regarding *regulation and oversight* and *strategic vision*, the participants identified several measures in place. These included yearly work plans with specific targets. The county and subcounty management teams supported the attainment of these targets by conducting meetings, providing support for the supervision of facilities, and discussing service provision progress with staff at the various facilities. Professional bodies provided standards and registered staff to ensure that high professional work standards were maintained. However, not all participants agreed:

***Yes, the targets are there, but they are not always followed or hon oured.***

(Male, 36–40 years)

The participants agreed that information was analysed by county and subcounty management teams in review meetings, for developing work plans, writing reports, identifying issues, and setting priorities. Respondents also indicated that information was used to allocate resources, such as budgeting for equipment, commodities, and drugs. Some participants thought information was used as a tool for transparency; others thought that it provided evidence for programmes:

***Interventions are derived from data.***

(Male, ≥41 years old)

***All reports generated are used to plan for the future.***

(Female, 26–30 years old)

There were contrasting opinions regarding the good use of information, however, and some participants thought that data were not being used appropriately. Respondents maintained that:

***Information procedures are not followed as they are supposed to be.***

(Female, 31–35 years old)

***The use of information is minimal. Mostly they use guidelines and information from national level.***

(Male, ≥41 years old)

***Occasionally, the information is followed, but sometimes it is not as it is supposed to be done.***

(Male, ≥41 years old)

***There is no clear way of passing information.***

(Female, 26–30 years old)

Participants reported that mechanisms were employed – involving community members, health facility in-charge, and political leaders – concerning the distribution of resources to ensure inclusion and equity. These mechanisms included waiver systems for poor people to ensure that they were not discouraged from seeking care for economic reasons, participatory budgeting, and the allocation of resources by political representatives (with devolution, members of the County Assembly – who are elected by the people – made decisions about resource allocation). Some participants, however, thought that these mechanisms were not clear.

According to participants, there were mechanisms in the county to ensure equity in resource allocation and access by marginalised people. The main way of including marginalised populations was during yearly participatory public budgeting activities. Sometimes, a member of the County Executive Committee on Health assessed the requirements of each facility. It was reported that all facilities were allocated money according to their budget. Several participants stated that the allocation of funds was based on population density, targets, and workloads at different facilities.

***We treat all people.***

(Male, 36–40 years old)

***[We include] all stakeholders in the budget [allocation].***

(Female, ≥41 years old)

Those who are considered marginalised, such as people with disabilities, reportedly did not pay for care when seeking treatment at the facility:

***Marginal communities like the disabled are exempted, or fees are waived when they seek medical services.***

(Female, 31–35 years old)

***There is a waiver system for the poor and paved roads to ease move ment for the disabled.***

(Male, 26–30 years old)

Participants reported that the facilities with which they were associated had 5-year strategic plans developed from the National Health Strategic Plan. Every year, annual operational plans were developed with set indicators, which were monitored monthly and quarterly by management teams. There were also audit departments that monitored the progress of the implementation of health policies and strategies to ensure set guidelines and procedures were followed. Reports were submitted, and meetings were held to facilitate the implementation of set activities. Additionally, various laws, regulations, and guidelines from the constitution, professional bodies, and the public service guided and regulated service implementation.

Respondents identified different ways in which accountability and transparency were achieved. Among these were having plans to guide performance, inviting different stake-holders to participate in the planning and implementation of activities, having professionals work, using receipts (accountable documents), following regulations like the Government of Kenya rules and procedures by introducing technology (computers and mobile phones). Participants emphasised:

***Staff are answerable at each level.***

(Female, 26–30 years old)

***Staff sharing of work stations …***

(Male, ≥41 years old)

***[Accountability] through audits***

(Male, 31–35 years old)

Some participants thought that procedures were not being followed, leading to comments on the “misappropriation of finances” and a belief that “accountability and transparency was poorly achieved in the county.”

## DISCUSSION

The purpose of this study was to examine the implementation of governance attributes as reported by decision makers of the Department of Health in Uasin Gishu County, Kenya. This was achieved by carrying out a desktop review and administering a semistructured questionnaire with 42 Likert-type questions and additional open-ended questions. The results suggest general dissatisfaction with the governance of health-care services in Kenya.

Most of the participants had a negative or neutral opinion about the implementation of governance attributes, with only one-tenth of the participants holding a positive view. Some of the reasons cited by the participants for poor implementation of governance were tribalism, corruption, conflicts of interest, inequitable sharing of resources, and political interference. Our findings differ from those of Mutale et al,^31^ who conducted research in Zambia and identified that 80% of participants thought that the implementation of governance attributes was good. One possibility for these contrasting results was that the Zambian study calculated mean scores generated from 4-point Likert scale responses, while our study established proportions. But at the same time, Gakuru et al argue that the Kenyan State has never been structured to represent or respond to the interests of the masses and the public good, but rather to serve the “interests of the incumbent political elite”.^40,41^ In Kenya and many other African countries, including South Africa, Botswana, Ghana, and Nigeria, governance is poor at the state level.^15,42,43^

The majority of participants thought that the implementation of accountability was poor, and two-thirds acknowledged that implementation of equity was poor. This suggests that accountability and equity have not been fully implemented in the context of health care in Uasin Gishu County. This contrasts with the findings of a study carried out in Ontario, Canada, where equity had been significantly implemented in primary health-care facilities. The health-care facilities had adopted physician services agreements, which incorporated different financial incentives that included bonuses. In Ontario, reporting requirements for physicians were voluntary and were limited to performance tied to incentivised tasks.^44^ In our context, further research needs to be undertaken to determine the factors that can improve equity in health-care facilities.

The use of information was also scored weakly among our participants. It can be deduced that when information and evidence are not clear to decision makers, there will be problems with transparency and accountability. Additionally, resources which were devolved were expected to be equitably distributed. Further, the Constitution of Kenya provides the legal framework to ensure more comprehensive, people-centred, and equitable provision of health services founded on a human rights approach.

The challenges of poor governance and lack of accountability in the use of public resources, including health, are not new.^45^ Kenya's Health Sector Strategic and Investment Plan (2015–2030) proposes a people-centred, more inclusive, and engaging approach to governance and proposes mechanisms to manage clients' issues and strengthen social accountability and stakeholder involvement. This implies that some challenges are being faced in the implementation of equity and accountability. According to the World Bank, when internal mechanisms of accountability are inadequate and fail to function, the situation calls for external accountability to ensure social accountability, that is, to ensure that the government is answerable for its decisions. This allows for constructive engagement between citizens and the government in monitoring the use of public resources for the purposes of service delivery, protection of rights, and community welfare protection. However, Cleary et al argue that internal bureaucracy interferes with external accountability.^19,22^

Notwithstanding this, there were positive perceptions from study participants about the implementation of elements related to *strategic vision* and *regulation and oversight* in the provision and administration of health care. This is not surprising because the participants were highly trained and had a good understanding of the factors contributing to the implementation of governance attributes. Based on the qualitative findings of the study, the participants demonstrated their understanding of governance as including a strategic vision, providing regulation and oversight, and being transparent and accountable. More research needs to be carried out to understand the effectiveness of the implementation of governance attributes. With the devolution of power to the county level, oversight and supervision were also devolved closer to the health-care facilities. This had the effect of increasing the focus on the performance of health-care workers and on the fact that they had to know the strategic direction and the regulations in place for effective service delivery. In this regard, our study findings were in concordance with the findings of Siddiqi et al who asserted that, in Pakistan, health service governance, participation, and consensus orientation were growing across the levels of assessment: local, regional, and national.^4^ Further research is required to assess how *participation and consensus orientation* will perform as the implementation of devolution continues to mature in Kenya.

### Limitations

Methodologically, our study sought to explore a complex concept of governance, but this complexity resulted in variability of understanding among the participants. As this study explored only Uasin Gishu County, the findings cannot be generalised. Furthermore, the number of decision makers in health was not large enough for complex analyses; generally, undertaking factor analysis requires a large sample size to make firm conclusions with confidence.^46^

Questionnaires that use Likert scales should include a mix of positive and negative statements.^36^ However, in this study, the outcome was good governance, and it was necessary to frame questions positively so that participants could assess the extent to which good governance was being implemented. The questions were framed to cover both rules-based and outcome-based indicators of governance to elicit perceptions on their availability and implementation. Perspectives on implementation of governance attributes for general health-care workers could further enrich available knowledge, and in the larger study from which this substudy emerged, the perspectives of general health-care workers were sought. Governance attributes are intertwined, and, therefore, other critical factors may have been masked by the attributes that were studied and others that were not. The current study attempted to categorise questions into groups for clarity and understanding.

## CONCLUSION

This study identified components of health-care governance within the study area that were functioning well at the time of data collection, particularly *strategic vision* and *regulation and oversight*. It can be concluded that devolution of oversight mechanisms closer to facilities in the new system of governance had been effective to that point in time and should be further harnessed. In contrast, *transparency* and *accountability* scored poorly. This means that the public and junior officers did not know how things were done by their senior officers. This in turn resulted in perceptions of nepotism and inadequate use of information. When government works are undertaken with transparency and office holders are held accountable, corruption and other vices are reduced. We conclude that the success of implementation of an attribute is dependent on the implementation of others. For example, there cannot be good implementation of accountability with poor implementation of transparency. If the health system is to achieve good overall governance, all governance attributes must be well implemented. Caution should, therefore, be taken in interpreting individual and overall scores. We have also illustrated that an expanded tool – with a wide range of clear questions to reduce the complexity of questions – serves to enhance good understanding by participants and provides a basis for measuring governance for other interested researchers.
